# Supplementary material for: Heterogeneity of CD8αα intraepithelial lymphocytes is transcriptionally conserved between TCRαβ and TCRγδ cell lineages
Source: Front Immunol. 2025 Aug 5;16:1637209. doi: 10.3389/fimmu.2025.1637209 (PMC12361183; doi:10.3389/fimmu.2025.1637209)

Supplementary figure 1

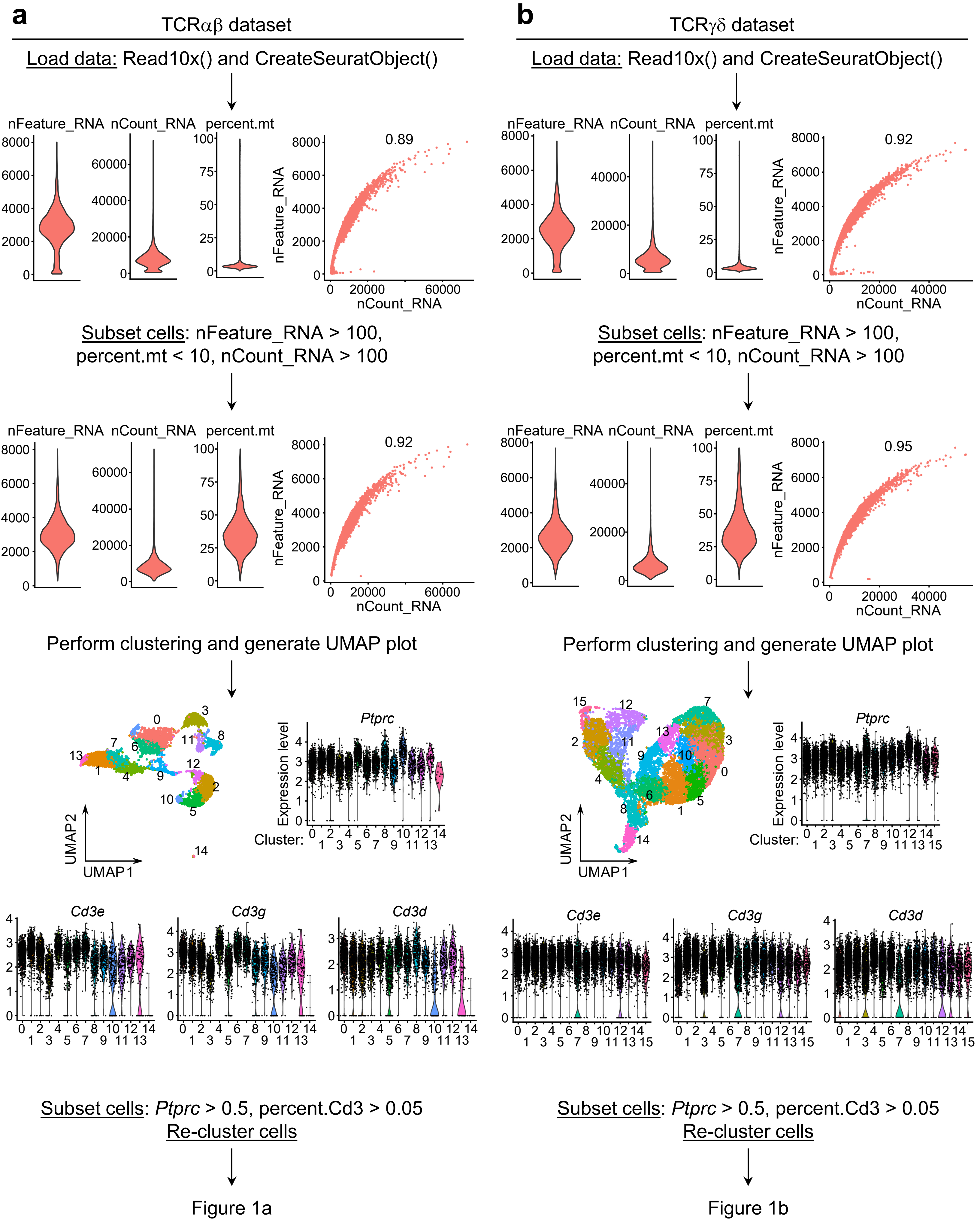

Supplementary figure 2

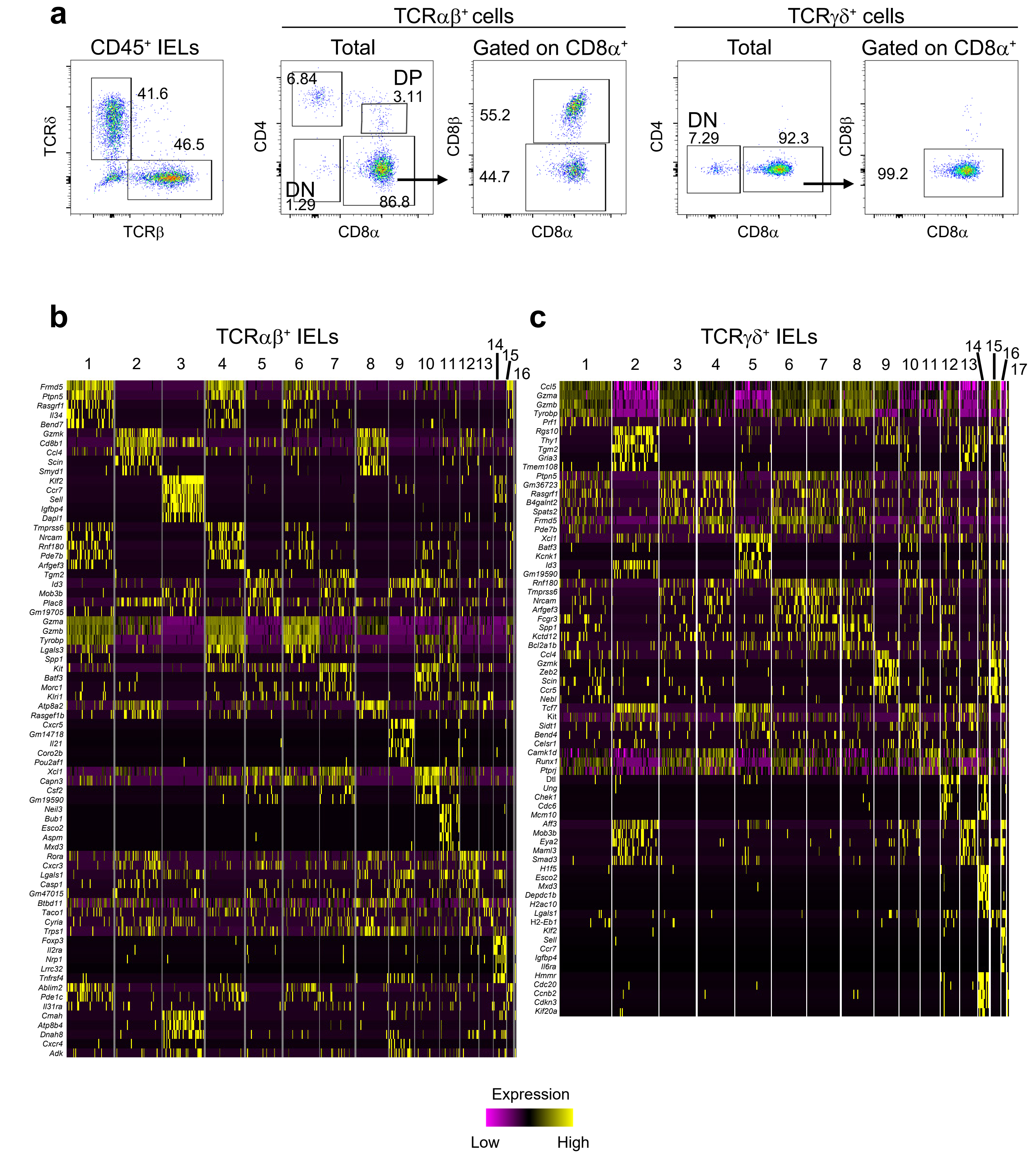

Supplementary figure 3

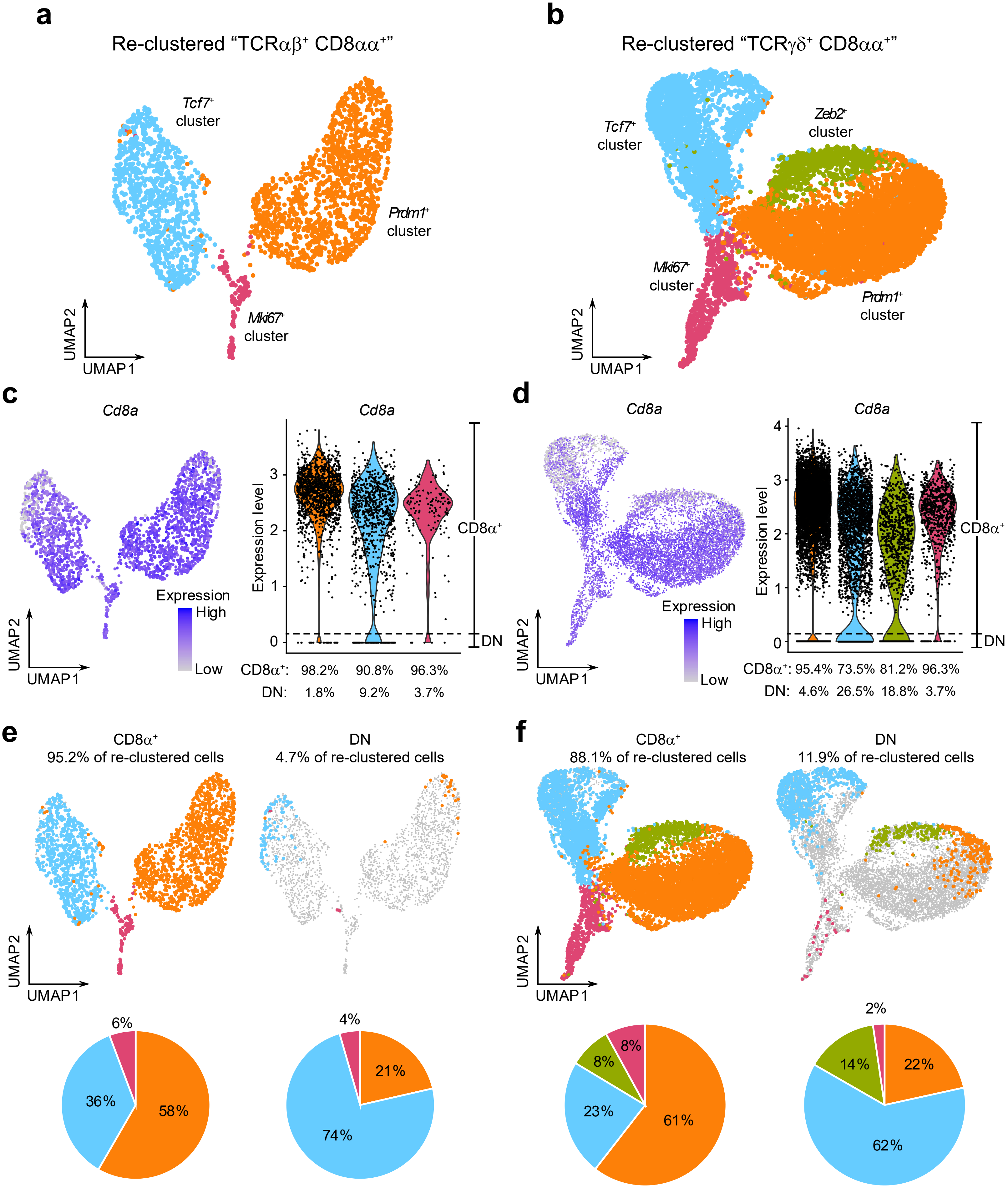

Supplementary figure 4

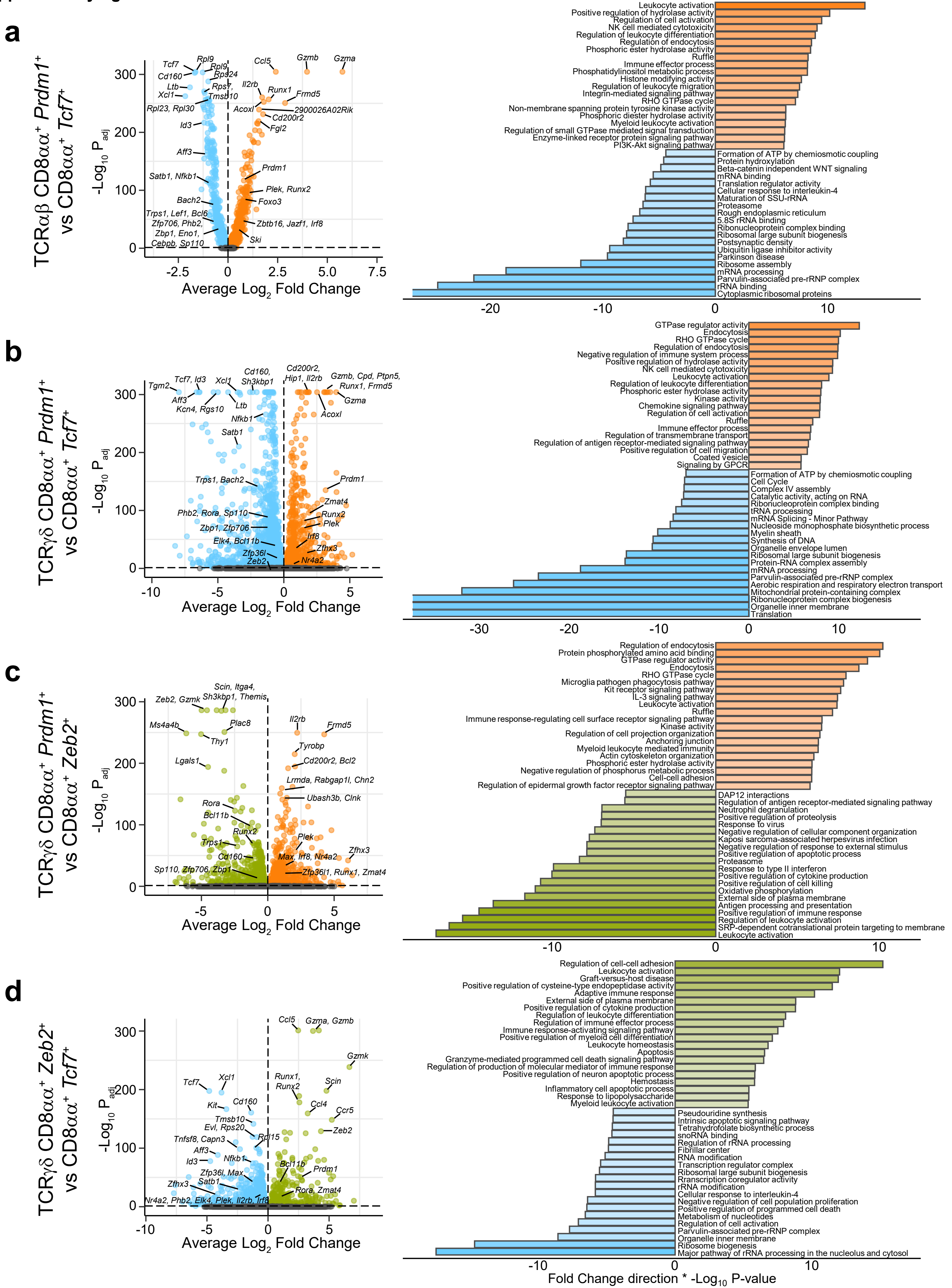

Supplementary figure 5

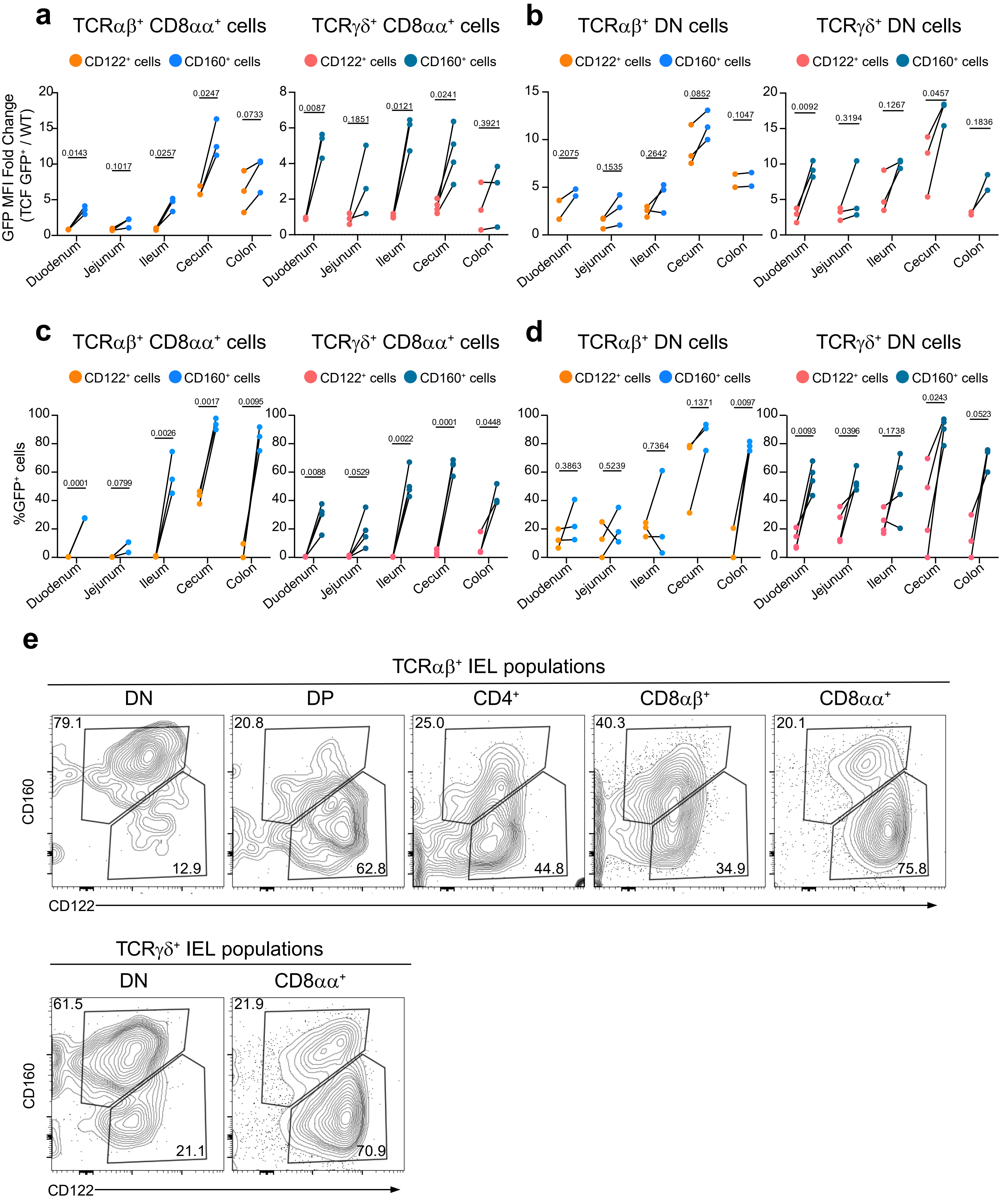

Supplementary figure 6

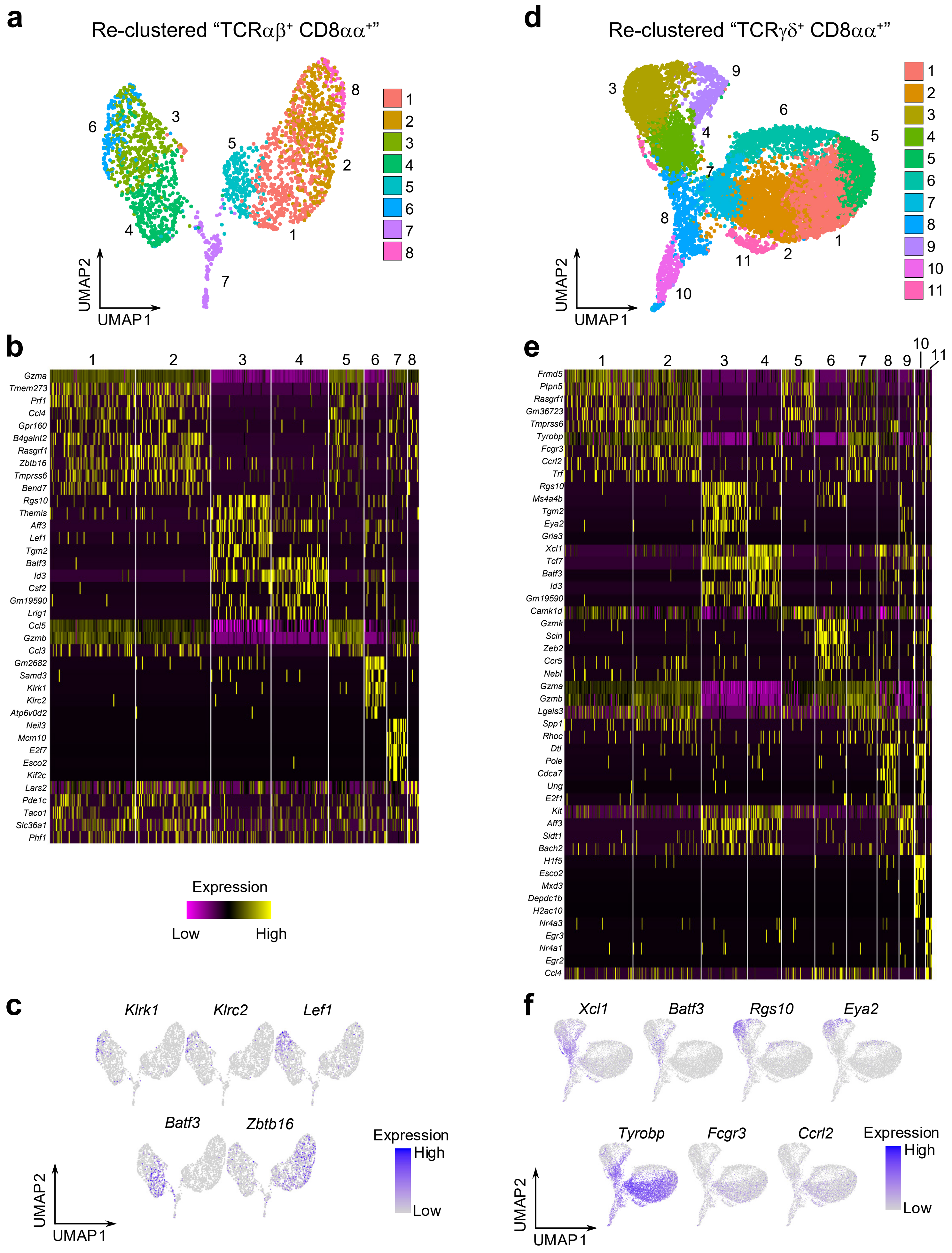

Supplementary figure 7

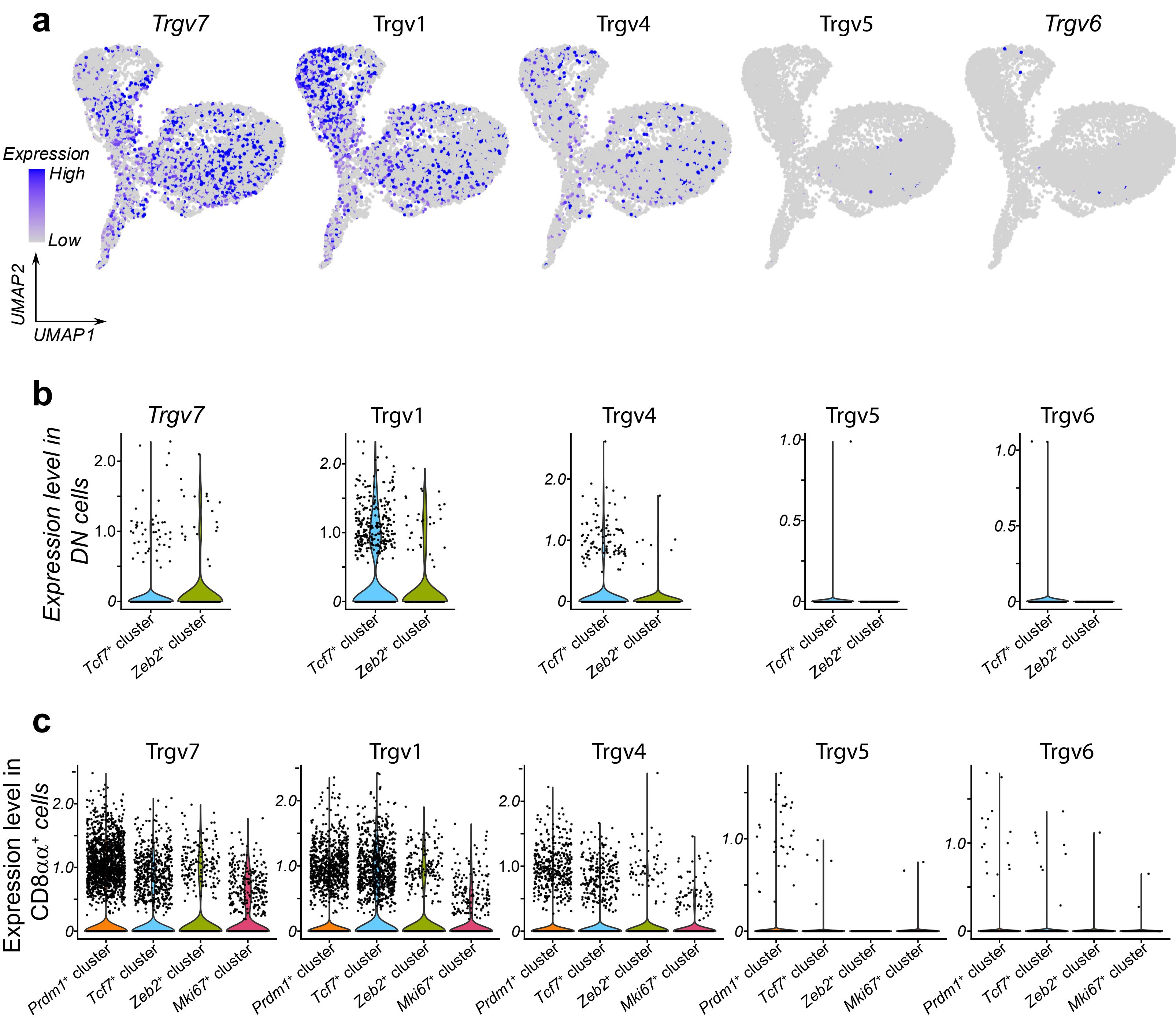

Supplementary figure 8

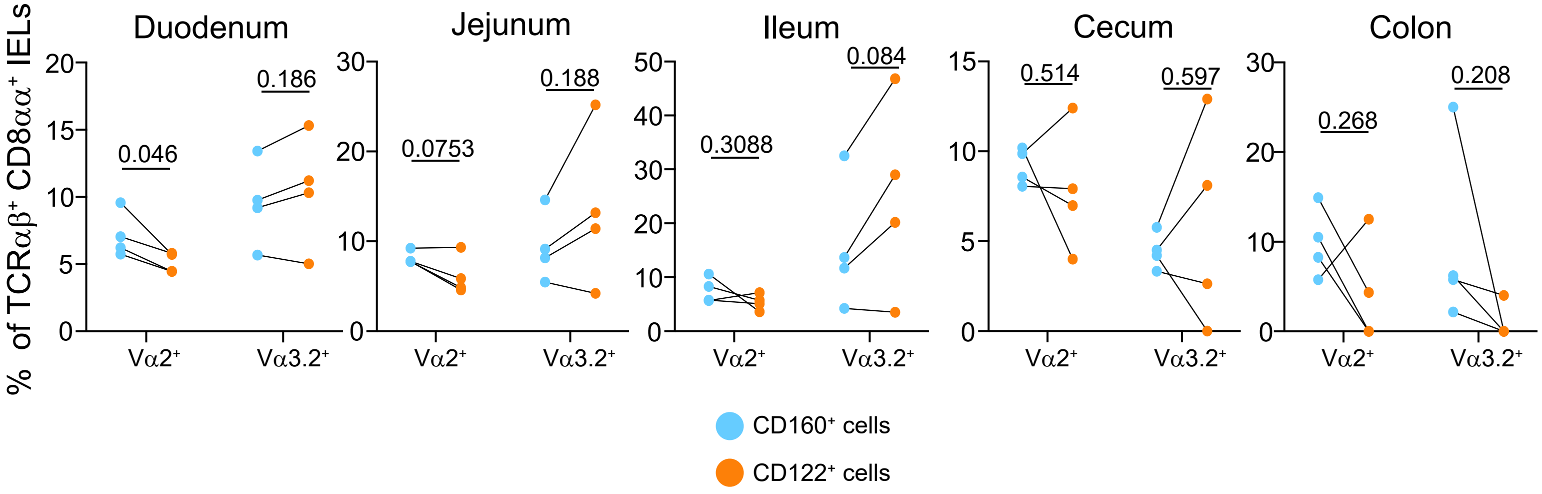

Supplementary figure 9

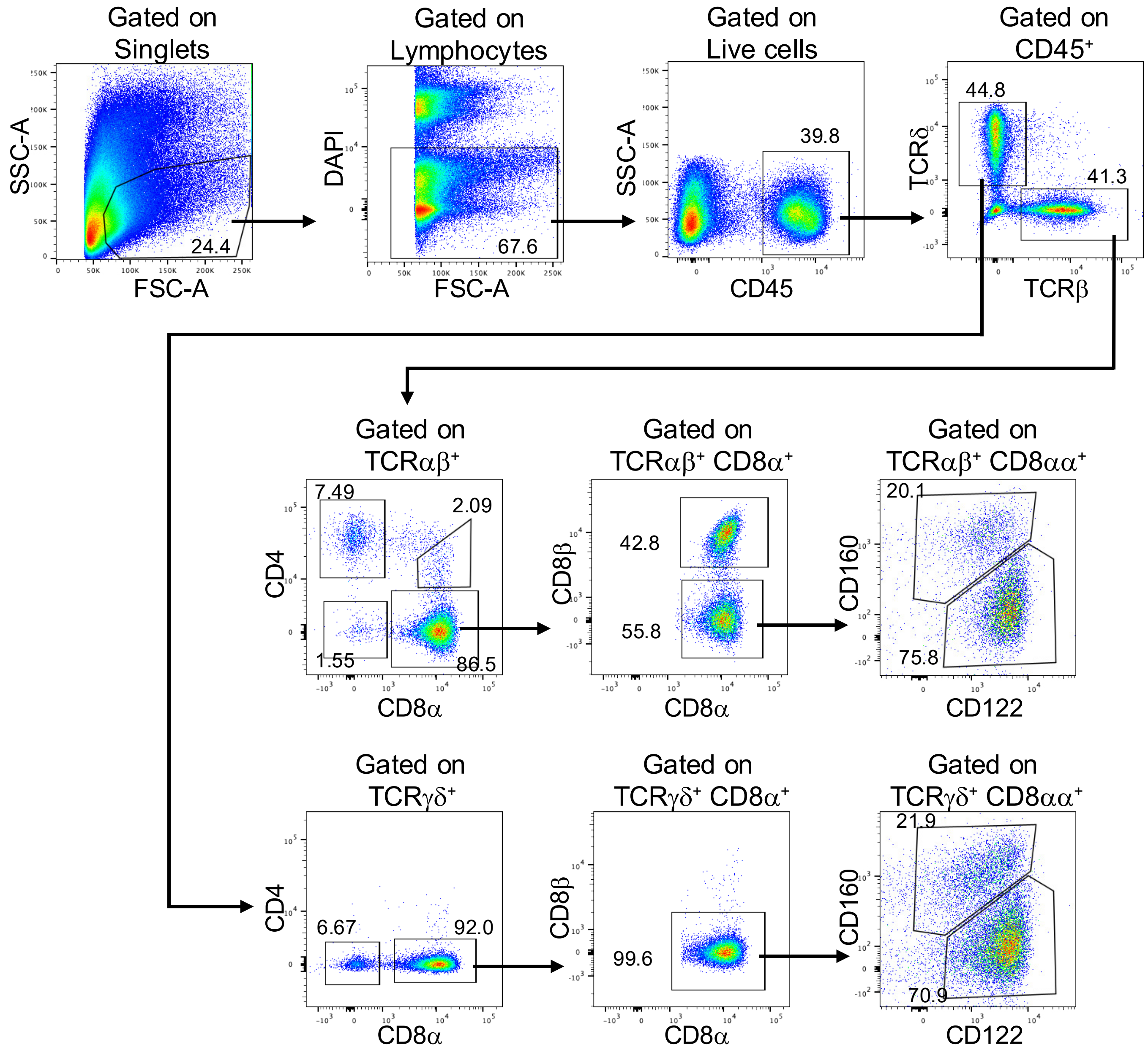

Supplement: Supplementary Figure 1 — (a) Preliminary analysis and quality control metrics for the TCRαβ+ scRNAseq dataset. (b) Preliminary analysis and quality control metrics for the TCRγδ+ scRNAseq dataset. [file Image1.pdf]
